# Poisoning in children and adolescents in Kermanshah city, Iran

**DOI:** 10.1186/s12887-024-04631-3

**Published:** 2024-02-22

**Authors:** Mitra Hemmati, Mohamad Reza Tohidi, Ali Mohammadi, Firozeh Jahanpour, Bahareh Andayeshgar, Sahar Fallah

**Affiliations:** 1 Department of Pediatrics, Associate professor of Kermanshah University of Medical Science, Kermanshah, Iran; 2 Clinical Research Development Center, Imam Khomeini and Mohammad Kermanshahi and Farabi Hospitals, Kermanshah University of Medical Sciences, Kermanshah, Iran; 3 Department of Health Information Technology, Paramedical School, Kermanshah University of Medical Sciences, Kermanshah, Iran; 4 Department of Biostatistics, School of Health, Kermanshah University of Medical Sciences, Kermanshah, Iran

**Keywords:** Intentional poisoning, Unintentional poisoning, Children, Adolescent, Iran

## Abstract

**Background:**

Poisoning among children and adolescents is a public health problem worldwide. To take preventive measures, the pattern of this problem should be determined. This study aimed to describe the demographic characteristics of poisoning in children and to investigate the relationship between the types of poisoning and demographic factors in children in Kermanshah province.

**Methods:**

This cross-sectional, descriptive-analytical study was conducted on 250 children and adolescents under 18 years of age who were referred to Mohammad Kermanshahi Pediatric Hospital in Kermanshah province due to poisoning during 2019–2022. The demographic and epidemiological data of patients were extracted from their medical files and analyzed.

**Results:**

Out of 250 cases of poisoning, 173 (69.2%) cases were unintentional, 96 (55.5%) of whom were boys. Further, 77 (30.8%) cases of poisoning were intentional, of whom 49 (63.6%) were girls. There was a significant difference between gender and intentional and unintentional poisonings (*p*-value = 0.005). The median age of unintentional poisoning was 3 (IQR = 2.5) and that of intentional poisoning was 14 (IQR = 2). Most cases of poisoning were in cities, 145 (83.8%) of them were unintentional and 66 (85.7%) were intentional. Most cases of intentional and unintentional poisonings occurred in spring 2017 (35.1%) and autumn 2016 (34.6%), respectively. The most common causes of poisoning were narcotics (*n* = 36, 34.3%) and drugs (*n* = 35, 33.3%) in the age group 0–3 years and drugs (*n* = 46, 66.9) in the age group 11–18 years.

**Conclusions:**

The most common causes of poisoning were narcotics and drugs in children and drugs in adolescents. To prevent poisoning in children, parents are required to increase their knowledge of the safe storage of narcotics and drugs, such as not storing methadone in a water bottle. Targeted evaluation and preventive measures are also needed in adolescent poisoning.

**Supplementary Information:**

The online version contains supplementary material available at 10.1186/s12887-024-04631-3.

## Background

Toxins are potentially harmful substances that can harm the human body [1]. Poisoning occurs when these toxins are ingested, inhaled, or absorbed through the skin, which can cause significant morbidity or mortality at high concentrations [2]. Poisoning has a significant impact on society in terms of health, economy, and culture [3]. Poisoning among children is a global public health problem and one of the leading causes of unintentional injuries in children [4–6]. It is also thought to be one of the main causes of disease in developing and developed countries. Poisoning accounts for about 2% of all deaths from injuries in developed countries and about 5% in less developed countries, which is generally associated with low morbidity and mortality [7–9].

Poisoning can be classified into two groups, intentional and unintentional. Young children are common victims because of their innate curiosity and exploratory nature. However, intentional poisoning is common among adolescents. Most intentional poisonings occur in developing countries, and a large number of cases lead to death due to the high toxicity of substances and the scarcity of medical centers [8, 10–12]. According to the report of the World Health Organization (WHO) in 2012, 193,460 deaths were due to unintentional poisoning worldwide, 84% of which occur in low- and middle-income countries [6, 13]. In addition, the highest rate of death due to unintentional poisoning is reported in children under 5 years of age, which is caused by their curiosity to taste or swallow harmful substances, and in adults over 55 years of age [14, 15]. In the United States, it has been estimated that more than 70,000 visits to the emergency department annually are for unintentional poisoning of children, most of which occur due to unsupervised drug use in children under 6 years. Fortunately, the vast majority of these cases do not result in significant damage [16].

The underlying causes of poisoning in different countries vary depending on local customs and beliefs, demography, the socio-economic status of the population in that region, and the level of education. In addition, the patterns of poisoning may be different according to the people’s age and gender [17]. Since children like to eat everything, ingestion is the most common route of poisoning in them, accounting for approximately 77% of cases. Further, because children are mostly kept at home, acute poisoning (90%) often occurs at home, which is mostly caused by various drugs and hydrocarbons such as petroleum, bleaching solutions, pesticides, insecticides, and cosmetics [10, 18–20].

In Iran, several poisonings have been reported in children and adolescents [10, 21, 22]. Epidemiological characteristics of poisoning in children vary in different countries and also the cities of a country. Therefore, knowledge of regional poisoning patterns can play an essential role in planning for the prevention, care, and treatment of patients. This study aimed to describe the demographic characteristics of poisoning in children and also to investigate the relationship between types of poisoning and demographic factors in children in Kermanshah province.

## Methods

This study is a retrospective and descriptive-analytical analysis based on hospital records available in Mohammad Kermanshahi Hospital. Mohammad Kermanshahi Hospital is an educational and affiliated hospital of Kermanshah University of Medical Sciences and is the only super-specialized children’s hospital in Kermanshah province. The population of this study is children and adolescents under 18 years of age who were referred to this hospital due to poisoning during 2019–2022. The Ethics Committee of Kermanshah University of Medical Sciences approved this study with the code (IR.KUMS.REC.1401.157).

Due to the fact that patients who have visited the emergency department of the hospital, a file is created for them, and their case code, first and last name, and the reason for the visit are recorded in the registers. After calculating the sample size, based on the following formula: by.

considering the poisoning rate in the age group of 6–12 years from previous studies [4] as *p* = 82.5, type one error rate α = 0.05 **(***Z* = 1.96) and precision d = 0.05 resulted 221 samples. $n = {{z_{1 - \frac{\alpha }{2}}^2p(1 - p)} \mathord{\left/{\vphantom {{z_{1 - \frac{\alpha }{2}}^2p(1 - p)} {{d^2}}}} \right.\kern-\nulldelimiterspace} {{d^2}}} = 221$, by referring to the registers and using the multi-stage sampling method, 250 cases were selected from among the cases of poisoning during the years 2019–2022. In this way, we first randomly selected some from the daily registers of the years 2019–2022, and in the next step, we randomly selected 250 samples from among the codes related to poisoning. Children were divided into age groups of 1–3 years, 3–6 years, 6–11 years, and 11–18 years. The substances leading to poisoning were divided into five categories: detergents, petroleum products, drugs, narcotics, and others (alcohol, castor oil, insect poisons, plants, cosmetic and health products, and gas). By studying the clinical records of the patients that were available in the hospital archives, individual and epidemiological information including the date of admission, number of days of hospitalization, gender, age, place of residence, year and month of poisoning, type of poisoning (intentional and unintentional), mortality and the type of substance leading to poisoning was extracted.

We also obtained additional information that was not recorded for 14 patients: the number of days of hospitalization, mode of reaching the hospital, interventions performed-including gastric lavage, length of hospitalization, Whether or not she was in the pediatric intensive care unit, and if a psychiatrist was consulted.

### Statistical analysis

We used IBM SPSS Statistics, Version 26.0 (IBM Corp., Armonk, NY, USA) to analyze the data. The Kolmogorov-Smirnov test was used to evaluate the normality of the data. To describe the quantitative data, the mean, standard deviation, the median, and the interquartile range (given the non-normality of the data) were used. Frequency and percentage were used to describe the qualitative data. Further, Chi-square, Fisher’s exact, and Mann-Whitney tests were used to analyze the data. Arc/GIS Desktop 10.8 software was used to display the geographical distribution of poisoning frequency based on age groups and the factors leading to poisoning in the neighborhoods of Kermanshah city. *P* < 0.05 was considered statistically significant.

## Results

### Demographic characteristics of patients

**Fig. 1:**
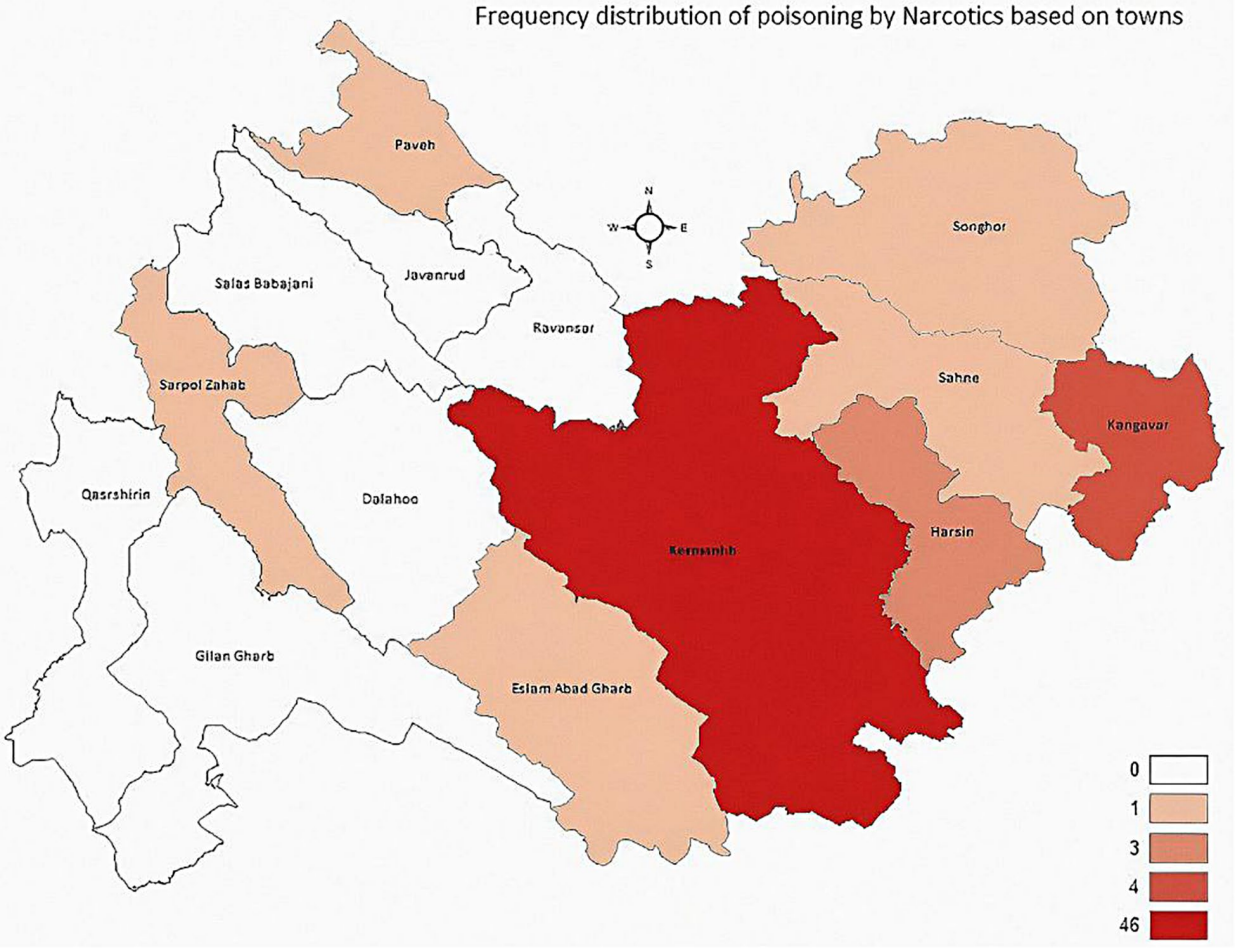
Frequency distribution of poisoning in towns

According to the results obtained from 250 cases of poisoning in children and adolescents under 18 years of age who were referred to Mohammad Kermanshahi Hospital during 2019–2022, 181 (72.4%) were from Kermanshah city, 44 (17.6%) were from Kermanshah towns, 18(7.2%) were from Kermanshah villages, and 7(2.8%) were from other cities of the country (Fig. 1). The number of girls and boys was almost equal, 126 (50.4%) girls and 124 (49.6%) boys. The youngest patient in this study was 1 year old and the oldest one was 17 years old. The mean ± standard deviation age of patients was 6.70 ± 5.37. The mean ± standard deviation age for girls was 7.49 ± 5.53 (the median age of girls was 5 years (IQR = 11.50)) and that of the boys was 5.91 ± 5.11(the median age of boys was 4 years (IQR = 11)) and there was a statistically significant difference between age and gender (*p*-value = 0.017). On the other hand, gender distribution in different age groups showed a statistically significant difference between age groups and gender (*p*-value = 0.036) (Tables 1 and 2).

**Table 1:** Distribution of age based on gender (median (IQR))

Variables		median (IQR)	*p*-value
Gender	Female	5(11.50)	0.017
	Male	4(8.75)	

**Table 2:** Frequency distribution of age category of poisoned children and adolescents based on gender

Gender	Age category				*p*-value
	1–3	3–6	6–11	11–18	
Female	49(38.9)	23(18.3)	6(4.8)	48(38.1)	0.036
Male	61(49.2)	23(18.5)	12(9.7)	28(22.6)	

**Fig. 2:**
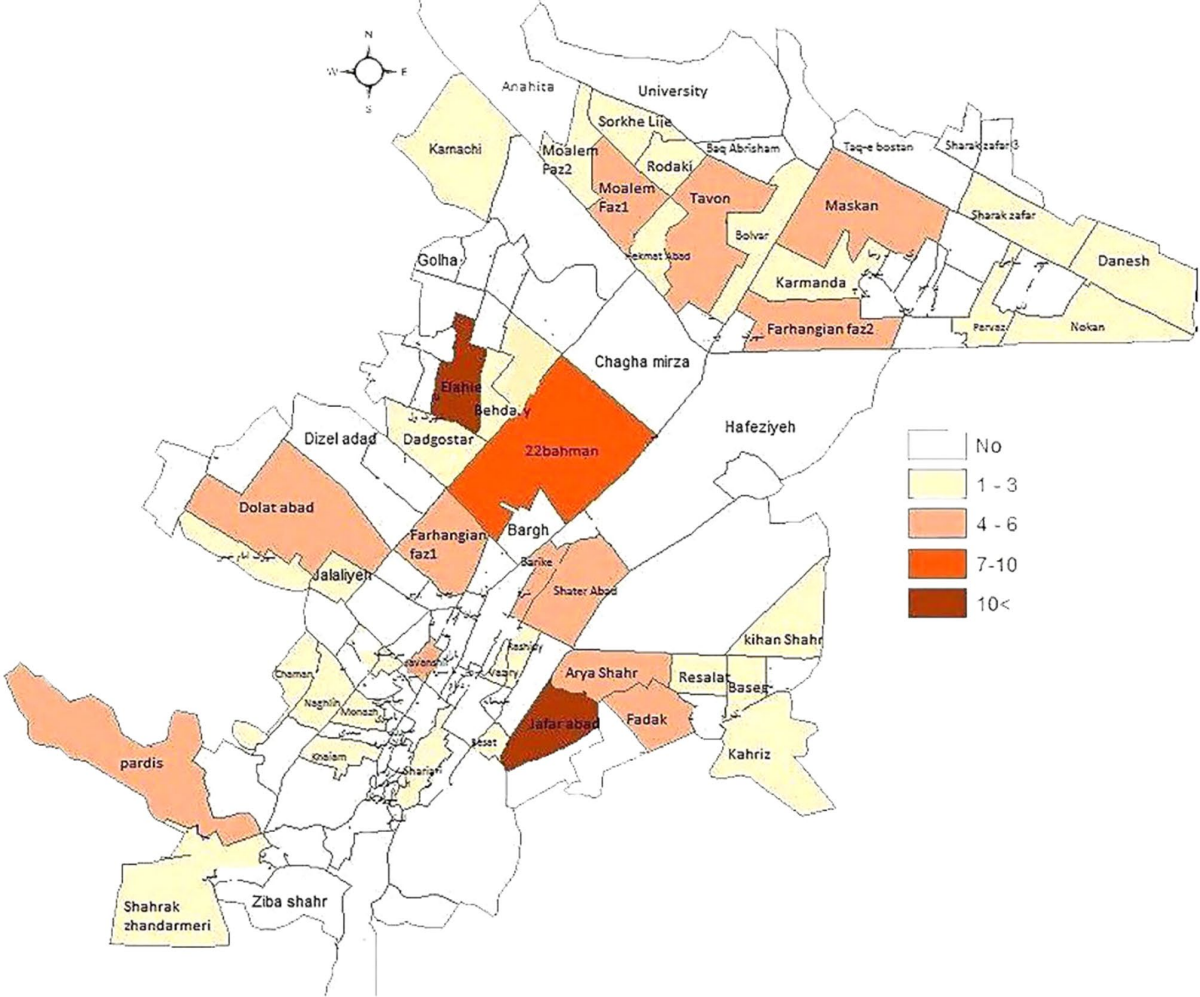
Geographical distribution of poisoning based on age group in the Kermanshah city

Of the patients, 110 (44%) were in the age group 1–3, 46 (18.4%) were in the age group 3–6, 18 (7.2%) were in the age group 6–11, and 76 (30.4%) were in the age group 11–18. The highest frequency of poisoning (*n* = 8, 7.3%) was in the age group 1–3 years in the Jafar Abad district, followed by the age group 11–18 years (*n* = 6, 7.9%) in the Elahieh district (Fig. 2). The highest frequency of patients’ birthplace was 185 (74%) in Kermanshah province. Among the different districts of Kermanshah city, the Elahieh district with 16 (6.4%) patients, and the Jafar Abad district with 15 (6%) patients had the highest frequency. Further, 211 (84.4%) patients lived in urban areas and 39 (15.6%) lived in rural areas. Of poisonings, 86 (34.4%) occurred in fall, 62 (24.8%) in winter, 55 (21.6) in summer, and 48 (19.2) in spring. Moreover, 106 (44.2%) drugs, 74 (30.8%) narcotics, 30 (12.5%) other substances, 19 (7.9%) detergents, and 11 (4.6%) petroleum products were among the poisoning causes in children and adolescents (Table 3). There were no fatalities due to poisoning.

**Table 3:** Demographic characteristics of poisoned children and adolescents in the study

Variable	Frequency	Percentage
Gender		
Female	126	50.4
Male	124	49.6
Age(year)		
1–3	110	44
3–6	46	18.4
6–11	18	7.2
11–18	76	30.4
Living area		
Urban	211	84.4
Rural	39	15.6
Season		
Spring	48	19.2
Summer	55	21.6
Fall	86	34.4
Winter	62	24.8
Cause of poisoning		
detergents	19	7.9
petroleum products	11	4.6
drugs	106	44.2
narcotics	74	30.8
other substances	30	12.5
gastric lavage		
Yes	157	66.5
No	79	33.5
Way of getting to the hospital		
independently	234	99.1
emergency medical team	2	0.9
psychiatric consultation		
Yes	54	22.9
No	182	77.1
PICU		
Yes	118	50
No	118	50

The most commonly performed treatment measures for the patient were serum therapy, drug interventions, and antidotes. Antidotes used included- naloxone (*n* = 72), activated charcoal (*n* = 144), sodium bicarbonate (NaHCO3) (*n* = 4), Amp Vitamin K (*n* = 1), N-acetylcysteine (*n* = 2) and Amp Calcium gluconate (*n* = 1). Most drugs used in the treatment of poisoning included Pantoprazole (85.7%), dextrose (75.3%), Magnesium hydroxide (60%), Heparin (50.9%), hydrocortisone (0.9%), and epinephrine (0.4%), along with drugs, such as Sodium chloride, potassium chloride, Adenosine and, Omeprazole.

Only 16 patients (6.4%) had a previous history of poisoning. The vast majority of patients traveled to the hospital independently (*n* = 234, 99.1%), while the emergency medical team was only called for 2 children (0.9%).

157 patients (66.5%) had to undergo gastric lavage, while 79 patients (33.5%) avoided it. In addition, 129 (55.1%) patients underwent Activated Carbon + Gastric lavage. Gastric lavage was required by 81 (68.5%) girls and 76 (65.5%) boys. Also, 43.3% of patients who required gastric lavage were in the age group of 1–3 years, 16.6% in the age group of 3–6 years, 9.6% in the age group of 6–11 years and 30.6% in the age group of 11–18 years. The relationship between gastric lavage and gender and age groups was not statistically significant. Medical interventions were performed for most of the patients (83.5%), such as electrocardiogram, electroencephalography, abdominal ultrasound, kidney ultrasound, gastroscopy, and CT scan. 82.5% of girls and 84.5% of boys required medical interventions. There was no statistically significant difference between interventions performed and gender and age groups.

Of all the patients, 54 (22.9%) required a psychiatric consultation, 41 of whom were girls and 13 of whom were boys. A significant difference was found between sex and whether a psychiatric consultation was performed (*p*-value<. 0001). Also, 39 (72.2%) patients were in the age group of 11–18 years. There was a statistically significant difference between age groups and whether a psychiatric consultation was performed (*p*-value < 0001).

The mean and standard deviation of the number of days hospitalized for the patients was 1.31 ± 1.50 (median 1 and IQR = 1), for girls, it was 1.24 ± 1.63 (median 1 and IQR = 1) and for, boys it was 1.37 ± 1.36 (median 1 and IQR = 1), which considering that the Mann-Whitney non-parametric test performs the test in terms of mean ranks, there is a statistically significant difference between the number of days of hospitalization and gender (*p*-value = 0.004).

Also, 118 patients were hospitalized in the pediatric intensive care unit (PICU).

### Demographic characteristics of patients for intentional and unintentional poisoning

Out of 250 cases of poisoning, 173 (69.2%) were unintentional, of whom 96 (55.5%) were boys. Moreover, 110 (63.6%) patients were in the age group 3 − 1, 46 (26.6%) in the age group 3–6, 12 (6.9%) in the age group 6–11, and 5 (2.9%) in the age group 11–18. Furthermore, 77 (30.8%) cases of poisoning were intentional, of whom 49 (63.6%) were girls; 6 (7.8%) cases were in the age group 6–11 and 71 (92.2%) patients were in the age group 11–18. There was a significant difference between gender and intentional and unintentional poisonings (*p*-value = 0.005) (Table 4).

**Table 4:** Frequency distribution of intentional and unintentional poisoning based on demographic characteristics

Variables		Intentional	Unintentional	*P*-value
Gender	Female	49(63.6)	77(44.5)	0.005
	Male	28(36.4)	96(55.5)	
Age	1–3		110(63.6)	< 0.001
	3–6		46(26.6)	
	6–11	6(7.8)	12(6.9)	
	11–18	71(92.2)	5(2.9)	
Leaving area	urban	66(85.7)	145(83.8)	0.702
	Rural	11(14.3)	28(16.12)	
season	Spring	27(35.1)	21(12.1)	< 0.001
	Summer	10(13)	44(25.5)	
	Fall	26(33.8)	60(34.6)	
	Winter	14(18.2)	48(27.71)	
gastric lavage		51(70.8)	106(64.6)	0.353
psychiatric consultation		40(55.6)	14(8.1)	< 0.001

The mean ± standard deviation age of unintentional patients was 3.50 ± 2.68(The median age of unintentional poisoning was 3 (IQR = 2.5)) and that of intentional poisoning was 13.89 ± 1.65(The median age of intentional poisoning was 14 (IQR = 2)). In unintentional poisonings, the mean ± standard deviation age of girls was 3.48 ± 2.59 (the median age of girls was 3 (IQR = 2)) and that of boys was 3.53 ± 2.77(the median age of boys was 2.5 (IQR = 3)). As for intentional poisonings, the mean ± standard deviation age of girls was13.79 ± 1.65(the median age of girls was 14 (IQR = 2)) and that of boys was 14.07 ± 1.68 (the median age of boys was 14 (IQR = 3)), indicating no significant difference between intentional and unintentional poisonings in terms of age (Table 5).

**Table 5:** Distribution of age based on gender in intentional and unintentional poisoning (median (IQR))

Variables		unintentional	*P*-value	intentional	*p*-value
age	female	3(2)	0.566	14(2)	0.521
	male	2.5(3)		14(3)	

Of poisonings in cities, 145 (83.8%) were unintentional and 66 (85.7%) were intentional. The highest frequency of intentional poisoning was 7 (9.1%) in the Elahieh district and 10 (5.8%) in the Jafar Abad district. In the Jafar Abad, narcotic drugs (*n* = 7, 70%) had the highest frequency in unintentional poisonings, and in the Elahieh, drugs (*n* = 4, 66.7%) were the most frequent in intentional poisonings (Fig. 2). The most frequent cases of intentional poisoning occurred in the spring of 2017 (35.1%) and fall of 2016 (33.8%) and the most frequent cases of unintentional poisoning happened in the fall of 2016 (34.6%). Table (4).

The mean and standard deviation of the number of hospitalization days for patients with unintentional poisonings was 1.29 ± 1.43 and for patients with intentional poisonings was 1.36 ± 1.65, that there is no statistically significant difference between the number of hospitalization days and intentional and unintentional poisonings (*p*-value = 0.166).

Gastric lavage was performed for 106(64.4%) patients with unintentional poisonings and 51(70.8%) patients with intentional poisonings. Also, 54.3% of patients with unintentional poisoning and 40.3% of patients with intentional poisoning were hospitalized in PICU.

Of 40 (55.6%) patients with intentional poisoning, psychological counseling was done 32(68.1%) of them were girls and 8(32%) were boys, and There was a significant difference between gender and the need for psychological counseling among intentional poisonings (*p*-value = 0.003). Also, counseling was done with the families of 14 (8.5%) patients who were accidentally poisoned, most of them were poisoned by drugs and medicine (78%).

### Frequency distribution of the type of substance leading to poisoning

Regarding the type of poisoning, 19 (7.9%) were caused by detergents, 11(4.6%) by hydrocarbons, 106 (44.2%) by drugs, 74 (30.8%) by narcotics, and 30 (12.5%) by other substances. The most common causes of poisoning were narcotics (*n* = 36, 34.3%) and drugs (*n* = 35, 33.3%) in the age group 1–3 years, and drugs (*n* = 46, 66.9%) in the age group 11–18 years. Also, the most common cause of poisoning was drugs in boys (*n* = 64, 53.3%) and girls (*n* = 42, 35%). Among unintentional cases, narcotics were the most frequent (*n* = 62, 37.1%), and among intentional cases, drugs were the most frequent (*n* = 49, 67.1%). There was a statistically significant difference between poisoning causes and each of the demographic characteristics (Table 6).

**Table 6:** Frequency distribution of the type of substance leading to poisoning based on demographic characteristics

Variables		type of substance leading to poisoning					*p*-value
		Detergents	petroleum products	Drugs	Narcotics	Other	
Age	1–3	13(12.4)	8(7.6)	35(33.3)	36(34.3)		< 0.0001
	3–6	3(6.5)	3(6.5)	19(41.3)	20(43.5)		
	6–11	2(11.8)	0	6(35.3)	5(29.4)		
	11–18	1(1.4)	0	46(66.9)	13(18.1)	0.007	
Gender	Female	4(3.3)	3(2.5)	64(53.3)	36(30)		0.007
	Male	15(12.5)	8(6.7)	42(35)	38(31.7)	0.013	
Living area	Urban	16(7.8)	7(3.4)	97(47.5)	63(30.9)		0.013
	Rural	3(8.3)	4(11.1)	9(25)	11(30.6)	< 0.001	
Type of poisoning	unintentional	18(10.8)	11(6.6)	57(34.1)	62(37.1)		< 0.001
	intentional	1(1.4)	0	49(67.1)	12(16.4)	11(15.1)	

## Discussion

Poisoning is a significant public health problem and it is one of the common reasons for referral to the emergency department of the hospitals [23, 24]. Most poisonings are seen in children and adolescents and approximately 0.5-2% of the reasons for applying to emergency outpatient clinics are in childhood [25, 26], on the other hand at least 500,000 people die annually from poisoning worldwide [27, 28]. Non-fatal poisoning is 20–30 times more prevalent and often causes long-term morbidities that severely reduce patients’ quality of life and put a strain on healthcare services and society worldwide [29]. This public health problem can be prevented with basic precautionary measures. on the other hand, due to the current social and economic conditions, exposure of patients to toxic substances is constantly changing, therefore, having information about the common types of poisoning and their causes in Different age groups will be useful.

In our study, out of 250 cases of poisoning patients referred to Mohammad Kermanshahi Hospital during 2019–2022, 126(50.4) were female, and 124(49.6) were male, which were almost equal in terms of gender distribution. The highest frequency of poisoning was in the age group of 1–3 years, 110 (44%) patients were in the age group 1–3 years, 46 (18.4%) in the age group 3–6 years, 18 (7.2%) in the age groups 6–11, and 76 (30.4%) in the age group 11–18.

According to the results of this study, most poisonings occurred in cities (*n* = 211, 84.4%), and in the fall season, 86 (34.4%). The most common causes of poisoning were drugs (*n* = 106, 44.2%) and narcotics (*n* = 74, 30.8%). There were no fatalities due to poisoning.

The most frequently administered antidotes were activated charcoal (144) and naloxone (72). Gastric lavage was applied to 157 patients (66.5%). In the pediatric age group, activated charcoal and gastric lavage remain the most frequently used treatments [25, 30]. The use of activated carbon and gastric lavage in children depends on the poisoning agent and the time elapsed between poisoning and the patient’s admission to the emergency department [25].

There were 77 intentional poisonings and 173 unintentional poisonings in this study. The highest frequency of unintentional poisoning was in the age group 1–3 years and it was higher in boys than in girls (*n* = 96, 55.58%). Exploratory swallowing by toddlers is a common reason for visiting a doctor [16]. Young children are naturally curious and explore their environment with all their senses.They tend to taste things and put them in their mouths as a result. During their development, they are not aware of potential dangers and are drawn to colorful items, such as bottles that hold household products and some medications. The behavior of adults, including the use of medicinal products, is often mimicked by preschool children. These factors together change the exploratory consumption leading to potential accidental poisoning into a common problem in this age group [29]. The higher prevalence in boys seems to be due to the nature of boys who are more active and adventurous than girls, however, the exact reason is unknown [4, 17]. In some studies, the highest frequency of unintentional poisoning has been reported in the age group under 5 years [4, 6, 16, 25] or 1–3 years [19, 23, 24, 31–33]. And in most studies unintentional poisoning was higher in boys than in girls [4, 22, 34, 35], yet in some studies was higher in girls [31, 36].

On the other hand, the highest frequency of intentional poisonings was in the age group 11–18 years (*n* = 71, 92.2%), which indicated a statistically significant difference between intentional and unintentional poisonings in age groups (*p*-value < 0.001). Also, the prevalence of intentional poisoning in this study was higher in girls (*n* = 49, 63.6), and there was a statistically significant difference between gender and intentional and unintentional poisoning so the prevalence of poisoning in girls increased with age (*p*-value = 0.005). Also, among the intentional poisonings in this study, psychological counseling was done for most of the girls (68.1%). Intentional poisoning in the adolescent age group can be caused by vulnerability to stress, failure, or disappointment in relationships and exams, as well as incompatibility with and inability to cope with family expectations. Adolescents are not emotionally stable and mature enough to bear intense mental or physical pressure [37]. On the other hand, intentional poisoning can be caused by significant psychological problems, depression, or substance abuse disorders [38]. This gender difference in intentional poisoning can be due to a higher rate of psychiatric problems, especially depression, more emotional crises in adolescence, premature puberty, and hormonal changes in girls [39]. Women in mental health crises want to attract public attention to their problems rather than act to take their own lives [40, 41]. In other studies, The highest frequency of poisoning was in the age group 15–19 years [42], 16–25 years [43], 6–11 years [22], 12–17 years [44], and 13–18 years [4], and the prevalence of intentional poisoning was higher in girls in most studies [42, 44–47].

In the current study, most intentional (*n* = 66, 85.7%) and unintentional (*n* = 145, 83.8%) poisonings occurred in urban areas, which is consistent with the results of the previous studies [19, 22, 34, 39, 48]. The increase in poisonings in urban areas can be associated with more child neglect by working mothers and more access to drugs, detergents, and chemicals in urban areas. Further, the population in urban areas is higher, so more referrals are made to hospitals as a result of poisoning [34, 39].

In addition, the frequency of unintentional poisoning was higher in autumn (34.4) 60, On the other hand, most intentional poisonings occurred in the spring and the least in the summer (35.1%) which showed a statistically significant difference between the type of poisoning and season (*p*-value < 0.001). In some studies, the prevalence of unintentional poisoning, like our study, was higher in autumn [19], and in others, it was in spring [44, 49]and summer [22, 39]. This discrepancy might be due to the geographical diversity and the incidence of envenomation [27]. and previous studies report that intentional poisonings occurred in spring [42], fall and, winter and, the least in the summer [47, 50]. which shows the reduction of suicide during the summer months coincides with a distancing from the stress of secondary education. Also, various studies have reported school and exam problems to stimulate adolescent suicide [45, 51, 52].

In the current study, the most common types of poisoning in the age groups 1–3 and 3–6 were Narcotics with a frequency of 36(34.3%) and 20(43.5%), respectively, and the most common type of poisoning in the age groups 6–11 and 11–18 was drugs with the frequencies of (*n* = 6, 35.3%) and (*n* = 46, 66.9%), respectively, indicating a statistically significant difference between age and type of poisoning (*p*-value < 0.0001). The most common type of poisoning in the age group under 7 in some studies was Narcotics [39] and in others drugs [4, 21, 22, 44] or household chemicals [53]. One of the reasons for easy access to drugs is neighboring countries such as Afghanistan and Pakistan, which use Iran’s strategic position to smuggle these drugs to Europe [15, 54]. Another reason for Narcotics poisoning in young age groups was the use of methadone, which was mistakenly placed and stored in water/drinking bottles by parents [55, 56]. Moreover, among the causes of drug poisoning, the availability of these products, due to the lack of safe storage tips and the fact that children may observe other family members taking the drug, and the development of imitative behaviors around the age of 2 years may somewhat explain the higher risk of drug poisoning in young children [4, 57].

The strength of this study is that Mohammad Kermanshahi Hospital is the only super-specialized children’s hospital in Kermanshah provinc, and in terms of the geographical location of Kermanshah city, people from many neighboring cities, villages, districts and provinces, refer to this center to receive specialized and sub-specialized medical services. However, the limitations of our study is that the study was retrospective and the data were manually recorded in the files, and due to the retrospective collection method, some data may be missing.

## Conclusion

According to the information obtained from this study, the most common cause of poisoning in children under 6 years old was narcotics, which is caused by the increase in drug abuse and easy access in society. Also, the most common cause of poisoning in the age group of 6–18 years was drugs, which requires increasing the knowledge and awareness of parents and healthcare workers about the principles of poisoning prevention.

More attention should be paid to expanding efforts to educate parents, especially addicted parents, about keeping poisonous products in safe places out of the reach of children. On the other hand, manufacturers can also produce household poisonous products in resistant containers with warning signs.

Overall, these types of studies tell public health officials to focus more on poisoning prevention efforts.

### Electronic supplementary material

Below is the link to the electronic supplementary material.

Supplementary Material 1

## Data Availability

The dataset used and/or analyzed during the current study can be downloaded through the link below. https://s30.picofile.com/file/8471980776/data_poisoning_final.sav.html.
